# Flexible and Portable Random Laser Devices: Integration of Electrospun Fibers and Doped Polymeric Substrates

**DOI:** 10.1021/acsomega.5c03313

**Published:** 2025-07-23

**Authors:** Leandro H. Zucolotto Cocca, André L. S. Romero, Luiza A. Mercante, Kelcilene B. R. Teodoro, Cleber R. Mendonça, Leonardo De Boni, Daniel S. Correa

**Affiliations:** † Photonics Group, Institute of Physics, Federal University of Goia’s, 74690-900 Goiânia, Goiás, Brazil; ‡ Photonics Group, Institute of Physics of São Carlos, University of São Paulo, 13560-970 São Carlos, São Paulo, Brazil; § Nanotechnology National Laboratory for Agriculture (LNNA), 564899 Embrapa Instrumentação, 13560-970 Sao Carlos, São Paulo, Brazil; ∥ Institute of Chemistry, 28111 Federal University of Bahia (UFBA) 40170-280 Salvador, Bahia, Brazil

## Abstract

The development of flexible optical and photonic devices has attracted growing interest due to their promising applications in optical sensing and light-emitting technologies. Among these, devices based on random lasers stand out for their simple fabrication and unique emission properties. However, despite advances using various scattering centers, the use of electrospun fibers as scattering centers for random laser emission is still limited. Here, we propose a novel random laser design by incorporating electrospun polyacrylonitrile (PAN) fibers as scattering centers within a flexible, portable polymeric substrate doped with rhodamine 6G as the gain medium. The resulting platform exhibits efficient random lasing with spectral narrowing below 10 nm [full width at half-maximum (fwhm)] and low excitation thresholds near 2 × 10^–4^ J. These results demonstrate a robust, easy-to-fabricate, and flexible random laser system with potential applications in optical sensors and photonic devices.

## Graphical abstract

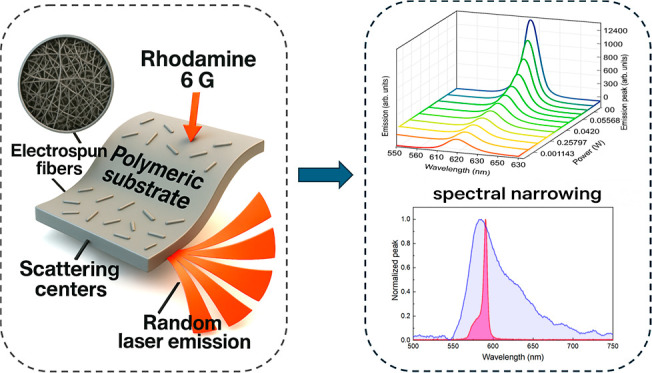

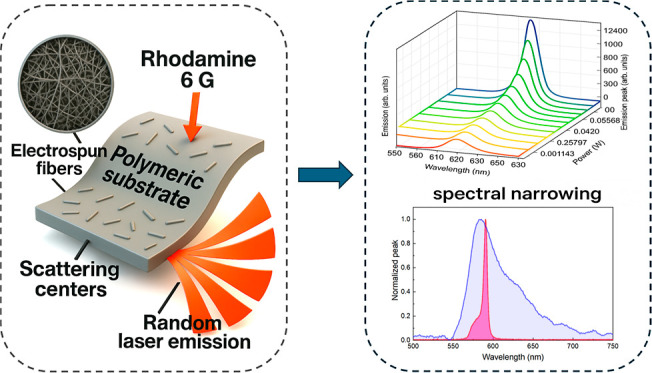

## 1 Introduction

In recent decades, significant efforts have been made in the search for flexible optical and photonic devices, driven by their potential for applications in optical sensing devices,^1–3^ light-emitting systems,^4,5^ and others.^6^ In this context, the optical phenomenon known as random laser^7,8^ can be employed in the manufacture and construction of photonic devices with simplicity and ease of fabrication.^9^ Basically, random laser emission arises from the combination of multiple scattering centers within a gain medium, usually based on organic dyes such as rhodamine.^10^ In contrast to conventional lasers, random lasers do not require mirrors in a resonant optical cavity to achieve the laser gain. Instead, the scattering centers in the gain medium contribute to the optical gain, resulting in emissions similar to those of conventional lasers, including spectral narrowing and an intensity threshold.^11–13^

As the optical feedback in random lasers is provided by light scattering, and the laser emission characteristics are influenced by them,^8,14^ random lasers can be utilized in the manufacture of optical sensors.^14^ For instance, studies have demonstrated the use of random lasers for biological sensing^15–17^ for quantifying fat concentration in milk^18^ and for sensing humidity,^19^ pH,^20^ and temperature.^21^

Random laser emission can be achieved using distinct types of scattering centers,^22^ including semiconductor powders,^23–27^ nanoparticles,^28^ and others.^29–37^ In addition, electrospun fibers can also serve as scattering centers in random laser emission.^22,38,39^ In fact, electrospun fibers have been extensively explored due to their broad applications across various fields of science and technology.^40,41^ Electrospun nanofibers can be composed of organic or inorganic materials^40,41^ and be manufactured with distinct shapes and morphologies.^22,40,41^ In addition, electrospun fibers present a high surface area/volume ratio and enhanced charge transfer properties, making them ideal for applications in nanophotonics and optoelectronics devices,^42^ as well as in sensors and biosensors.^40,41^ Another remarkable property of electrospun fibers is their highly random structure,^43^ which is also suitable for designing random laser devices.

In this context, here we aim to investigate random laser emission using electrospun fibers as the scattering center. While previous studies have employed electrospun fibers for this purpose,^38,43–47^ here we introduce a novel approach that leverages portable and flexible polymeric substrates doped with a dye (rhodamine 6G) to serve as the gain medium. Specifically, the electrospun fibers were deposited on top of these polymeric substrates, serving as scattering centers to enhance random laser emission from the polymeric substrate, as illustrated in Figure 1. The main goal was to verify if this approach can result in an easy-to-handle and mechanically flexible platform capable of generating random laser emission for optical and photonic devices.

**Figure 1:**
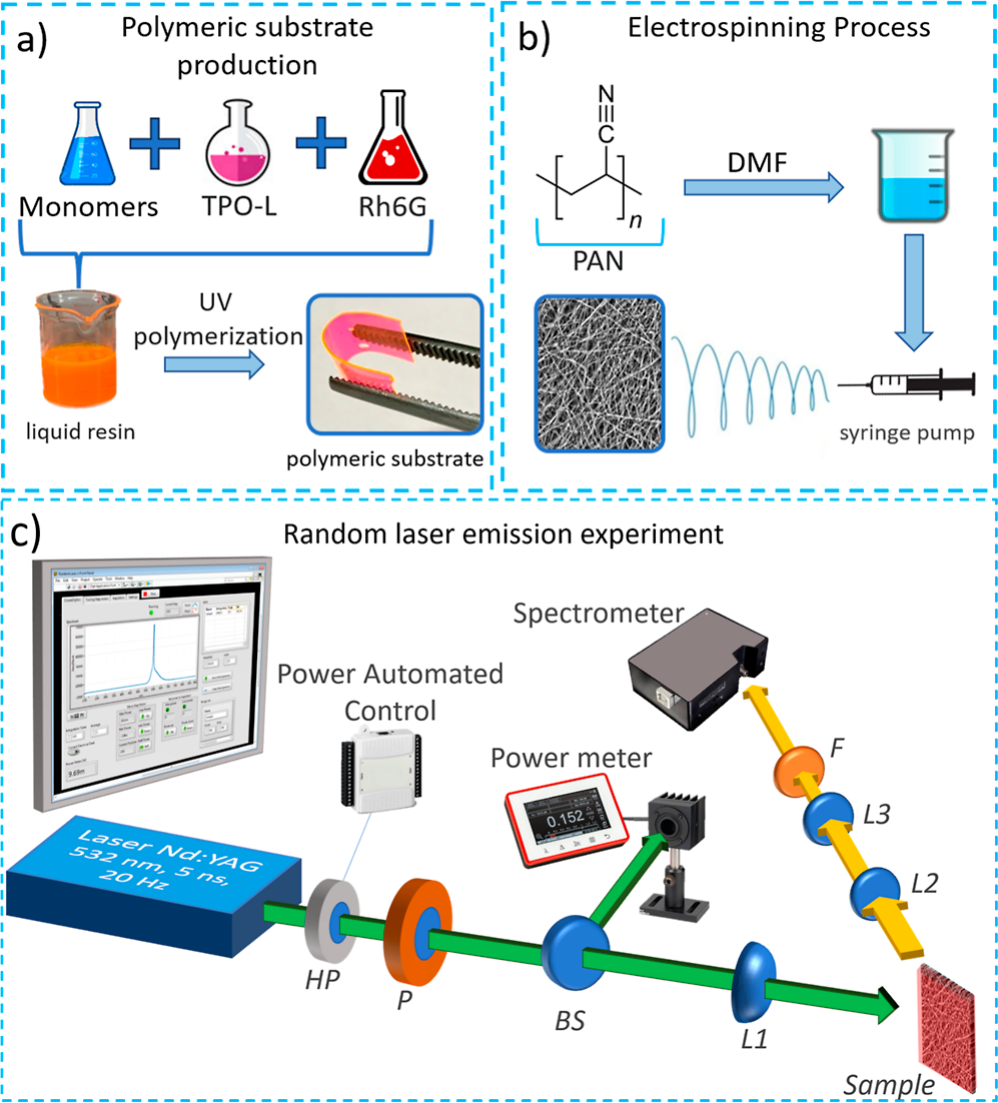
(a) Schematic representation of the manufacturing process of polymeric substrates doped with rhodamine. Initially, a mixture of two polymers (SR368 and SR499) is prepared and combined with the photoinitiator (Lucirin TPO-L). Next, the gain medium is added using a solution of rhodamine 6G in ethanol. After mixing, the solution is poured onto a microscope slide. To ensure uniform shape and thickness, another microscope slide is placed on top of the solution, resulting in flexible doped platforms with a thickness of approximately 200 μm. (b) Schematic representation of the electrospinning technique used for producing electrospun PAN fibers. (c) Setup for the automated random laser emission experiment. HP is the half-plate, P represents the polarizer, BS represents the beam splitter, L1 is the convergent lens that focuses the laser beam on the sample, L2 and L3 are the convergent lenses which focus the random laser emission to the spectrometer, and F is the 532 filter.

## 2 Materials and Methods

### 2.1 Sample Preparation

To prepare the polymeric substrate on which the electrospun fibers were deposited, two polymers were employed: tris­(2-hydroxyethyl)­isocyanurate triacrylate (SR368, Sartomer) and ethoxylated (6) trimethylolpropane triacrylate (SR499, Sartomer), in a 50 wt % ratio (1 g each). These two polymers were selected for constructing the polymeric substrate owing to the fact that both polymers possess trifunctional acrylate monomers that enable extensive cross-linking during the photopolymerization process.^48^ This is achieved by synthesizing a polymeric substrate that has increased mechanical strength and stiffness^48,49^ when compared to other polymeric compounds that may contain only monofunctional or nonfunctional acrylates, such as methyl methacrylate or butyl acrylate. Moreover, SR368 and SR499 monomers are able to form polymer substrates that can contain active compounds, providing the final substrate with functional properties, such as fluorescence or lasing characteristics. Specifically, here, these monomers were modified with rhodamine 6G, which will work as a gain medium aiming at laser action.

For initiating UV photopolymerization, 60 mg of the photoinitiator ethyl 2,4,6-trimethylbenzoylphenyl phosphinate (Lucirin TPO-L, BASF) was mixed into the polymer mixture. The solution was mixed for 2 h, and 1 mL of rhodamine 6G ethanolic solution (1.23 mg mL^–1^, equivalent to 2.5 × 10^–3^ mol·L^–1^) was added to the polymeric mixture, resulting in a final rhodamine 6G concentration of 0.061% wt. Subsequently, the polymeric solution was then heated until the ethanol evaporated, followed by exposure to UV radiation for 30 min to initiate the photopolymerization process. Finally, the polymer substrate was sliced into pieces of approximately 3 cm × 3 cm, with a thickness of 200 μm. Figure 1a illustrates the method employed to obtain the polymeric substrates doped with rhodamine 6G, which should work as a laser gain medium and also serve as flexible and easy-to-handle platforms for deposition of the electrospun fibers.

Micro/nanofibers were produced using the electrospinning technique^50,51^ due to its simplicity and cost-effectiveness. The electrospinning setup involves the following main components: a high-voltage source, a syringe containing the polymer solution that will form the electrospun fibers, a spinneret connected to the syringe and the high-voltage source, and a grounded metal collector.^52^ For the production of the electrospun nanofibers, a polyacrylonitrile (PAN) solution in *N*,*N*-dimethylformamide (DMF) (10% w/v) was used, as illustrated in Figure 1b. The PAN solution was loaded into a syringe, and the distance between the spinneret and the drum collector was set at 12 cm. The applied voltage was 12 kV, and the injection rate was maintained at 0.5 mL/h. Aiming to facilitate the fiber deposition and preserve its properties, the fibers were electrospun directly onto the polymeric substrates. For this, bare substrates were attached to the drum collector, which was kept under a constant rotation of 180 rpm. It should be mentioned that, prior to fiber collection on the substrate surface, a 30 min preliminary spinning was carried out to stabilize the electrospinning setup. Six samples of polymeric substrates coated with the electrospun fibers were obtained for evaluation of the electrospinning time, namely, 0, 10, 20, 40, 50, and 60 min. These sample platforms were designated as FB0, FB10, FB20, FB40, FB50, and FB60, respectively. Additionally, it should be emphasized that sample FB0 (the polymeric substrate without fibers) corresponds to the standard platform. The electrospinning procedure was performed at a temperature of 25 °C and humidity of nearly 35%.

#### 2.1.1 Substrate Characterization

The polymer platforms containing the electrospun nanofibers were characterized in terms of their morphology and chemical composition. The distribution of nanofibers over the fluorescent polymeric substrates was investigated by fluorescence microscopy, using an Olympus BX63F3 microscope, using a 100x lens and UV excitation at 360–370 nm. The morphologies of the coated substrates, as well as the interface substrate/nanofibers, were analyzed using a PHILL-IPS-XL30 FEG-SEM microscope operating at 2 and 3 kV. The samples were coated with platinum using a sputter coater (Leica EM SCD050). The structural morphologies of neat substrates and those modified with nanofibers were investigated with scanning electron microscopy (SEM), using a TM-3000 Hitachi microscope operating at 5 kV. The NF diameters were estimated using ImageJ software. In this procedure, 300 fibers were randomly selected from the SEM image, and their diameters were determined by using the aforementioned software. The chemical composition of neat and modified substrates and the interaction between components were evaluated by Fourier transform infrared spectroscopy (FTIR). For FTIR analysis, a Spectrum 1000 PerkinElmer spectrometer (software Spectrum), equipped with attenuated total reflection apparatus (ATR), was used, form which spectra were obtained in absorbance mode in the range from 4000 to 400 cm^–1^, using 32 scans and a resolution of 2 cm^–1^.

### 2.2 Random Laser Setup

A pulsed laser Nd:YAG (model Surelite I-20, Continuum), with a 20 Hz repetition rate, 5 ns pulse width, and frequency doubled to 532 nm with 100 mJ of maximum energy, was employed in order to excite the random laser emission platforms. Initially, the laser beam goes through a plate computer-controlled stepper motor coupled to a rotating carrier containing the 532 nm half-wave blade and a polarizer. A beam splitter is positioned at 45° after the polarizer to reflect 5% of the beam onto a power meter (Thorlabs). All of these components aim to control and acquire the pump power that will reach the random laser platform. A 10 cm convergent lens was used to focus the excitation light onto the polymeric platform, and the emitted radiation was collected in reflection mode at approximately 45° via a multimode optical fiber. The emission spectrum was acquired by using an Ocean Optics 2000 spectrometer. To block 532 nm excitation from reaching the spectrometer, an optical color filter was employed. All the equipment is connected to a computer and customized LabVIEW software, which was used to automate the experiment. This software provides mathematical tools to fit a Gaussian function to each emission spectrum and values such as the threshold energy and full width at half-maximum (fwhm) narrowing as a function of pump energy. More details about the experimental setup can be found in ref 53, and a scheme for the setup is shown in Figure 1c.

In summary, a fully automated experimental setup was employed in which beam shutters were included to ensure that the sample was exposed to the laser beam only during the data acquisition intervals. By this precaution, unnecessary exposure was minimized, and the sample’s optical integrity was preserved throughout the measurement process. It should also be emphasized that the stability of the pump laser, combined with the use of a custom-developed automated acquisition program, was found to have contributed positively to the measurements. Through this setup, precise control over acquisition timing and energy delivery was achieved, ensuring that consistent and reliable data collection was maintained without the variability introduced by external or manual factors.

## 3 Results and Discussion

### 3.1 Physicochemical Characterization of the Polymeric Substrate

Figure 2a shows the digital pictures of the neat and modified substrates fabricated after 10, 20, 40, and 60 min of electrospinning. A noticeable “whitening” effect is observed with increasing electrospinning time, resulting from the deposition of more nanofibers and the thickening of the film onto the substrate surface. Fluorescence microscopy micrographs further illustrate the extent of coating on substrates modified with nanofibers. Figure 2b–d show representative regions of the samples FB10, FB40, and FB60, respectively. Fiber-like structures can be seen covering the brighter regions corresponding to the fluorescent substrate, with their density increasing as the electrospinning time evolves. Figure 2e–g show FEG-SEM images of the corresponding samples analyzed by fluorescence microscopy. By this technique, it is also possible to verify the coating of the substrate with a thicker layer of nanofibers as a result of increasing the time of electrospinning.

**Figure 2:**
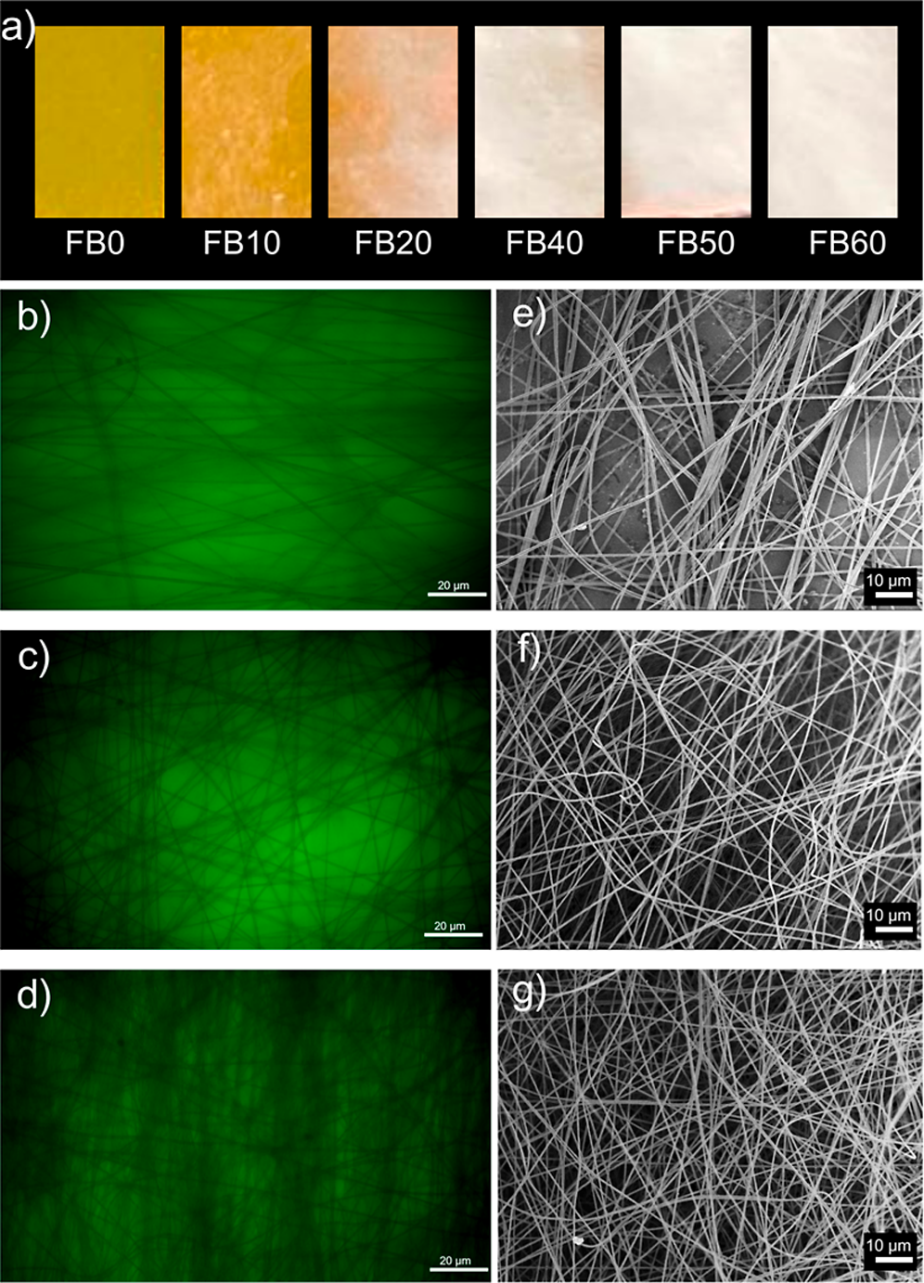
(a) Digital images of the substrates exposed to different times of electrospinning (0 to 60 min). Micrographs of fluorescence microscopy obtained for samples (b) FB10, (c) FB40, and (d) FB60. FEG-SEM micrographs of the samples (e) FB10, (f) FB40, and (g) FB60.

Figure S1a,b presents SEM images of the polymeric substrate without fibers. These images reveal that the substrates are free of grooves, cracks, or any other structural defects. Figure S1c displays the interface between the polymeric substrate and the layer of fibers in the sample FB60.

The composition of the platforms and the interactions between the components were characterized by the FTIR technique. The comparison among the FTIR spectra of the neat polymeric substrate and the modified substrate (sample FB60) is displayed in Figure S2. The FTIR spectra of PAN nanofibers were also recorded for comparison. It is observed that the FB60 spectrum is largely composed of the overlay of FB0 and PAN spectral bands. The FTIR spectra of NF and FB60 samples are similar, majorly expressing the PAN characteristic peaks, whose bands at 2938 cm^–1^, 2240 cm^–1^, and 1453 cm^–1^ are related to the elongation vibrations of the −C–H bonds of the CH_2_ groups, the −C≡N bonds, and the bending vibration of the −C–H bonds, respectively.^54^ Sample FB0, composed of the substrate without nanofibers, shows characteristic peaks of −CH_3_ and −CH_2_ observed at 2956 cm^–1^ and 2873 cm^–1^, respectively. In addition, −C=C– bonds and carbonyl groups, typically from acrylate groups, can be seen at 1595^–1^ and 960 cm^–1^ and 1670 cm^–1^ and 1730 cm^–1^, respectively.^55,56^

Scanning electron microscopy (SEM) images were collected for the produced electrospun fibers deposited onto the polymeric substrate, aiming at determining their diameters. Figure S3 shows SEM images of the FB20 sample, which is also typical for the other samples, once all electrospinning conditions were kept the same, but the collecting time was changed. The analysis (inset, Figure S3) revealed the formation of randomly oriented fibers with a smooth, nonporous surface, with an average diameter of 800 nm. As expected, the presence of a random 1D cylindrical structure provides a suitable condition for light propagation and scattering.^57,58^

### 3.2 Random Laser Experiments

The combination of the polymeric substrate and electrospun fibers (named random laser emitting platforms) was subjected to varying excitation powers to study their emission properties, as shown in Figure 3a for FB10 (Figure S4, in the support information, presents the emission spectra for FB0, FB20, FB40, FB50, and FB60). This study was performed to determine the random laser threshold and evaluate spectral narrowing (assessed via the fwhm). All six random laser-emitting platforms were exposed to identical experimental conditions at room temperature. In addition, it should be highlighted that the fibers were not directly exposed to the pumping laser; instead, the polymeric substrate itself was subjected to pump excitation in order to avoid any damage to the fibers. Additionally, it should be noted that the maximum laser power utilized did not damage either the fibers or the polymeric substrate.

**Figure 3:**
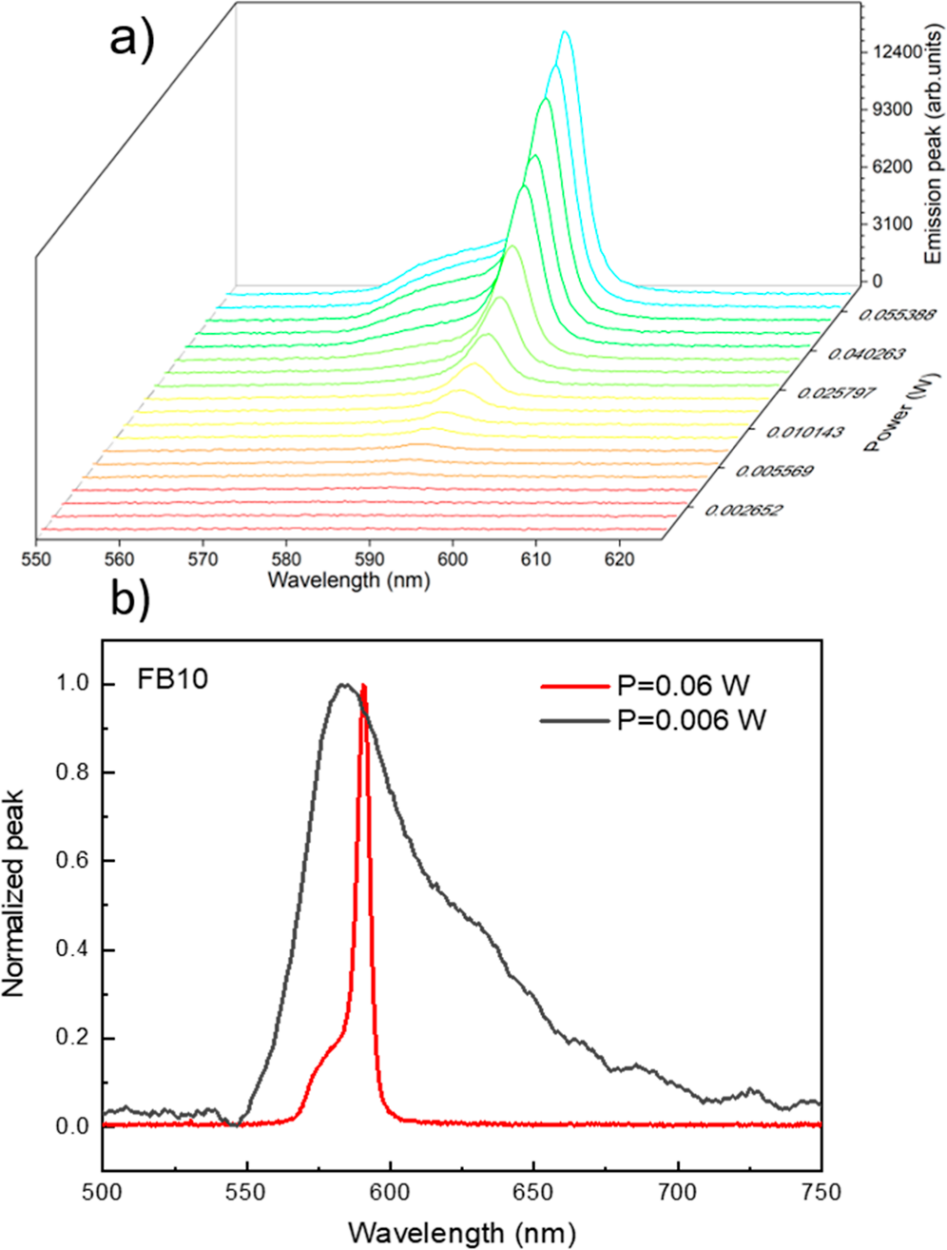
(a) Emission spectra as a function of pump power for FB10. (b) Emission spectrum for the sample FB10. The black line represents the emission spectrum under low excitation power (fluorescence/spontaneous emission regime), while the red line corresponds to the emission spectrum when the platform is excited with powers exceeding the laser threshold (laser regime).

Figure 3b shows the emission from FB10 in a spontaneous emission regime (black line, *P* = 0.006 W) and in a random laser regime (red line, *P* = 0.06 W). The spontaneous emission regime and laser regime for FB0, FB20, FB40, FB50, and FB60 are exhibited in Figure S5. Considering low pump powers (∼0.006 W), i.e., in the spontaneous emission regime, it can be observed from Figure 3b (black line) that the emission spectrum obtained is similar to that of rhodamine 6G in aqueous solution,^53^ exhibiting an fwhm of approximately 40–50 nm within the spectral range from 550 to 750 nm. This suggests that the presence of fibers does not significantly affect the spontaneous emission of rhodamine 6G. However, with increasing pump power, a narrowing of the fwhm is observed, and the laser regime is achieved, as shown in Figure 3b (red line).

Figure 4 illustrates the emission intensity and fwhm as a function of the pumping energy for sample FB40 (the emission intensity and fwhm for the other random laser platforms are shown in Figure S6. Figure 4 clearly shows the presence of a laser threshold at approximately 1.9 × 10^–4^ J for sample FB40. The presence of a threshold, observed in both the emission intensity and the fwhm, indicates the transition from the spontaneous emission regime to the laser emission regime. The results demonstrate that polymeric substrates doped with rhodamine 6G (gain medium) and the electrospun fibers (scattering center) deposited onto the polymeric substrates synergistically form a portable and easy-to-handle platform capable of producing random laser emission.

**Figure 4:**
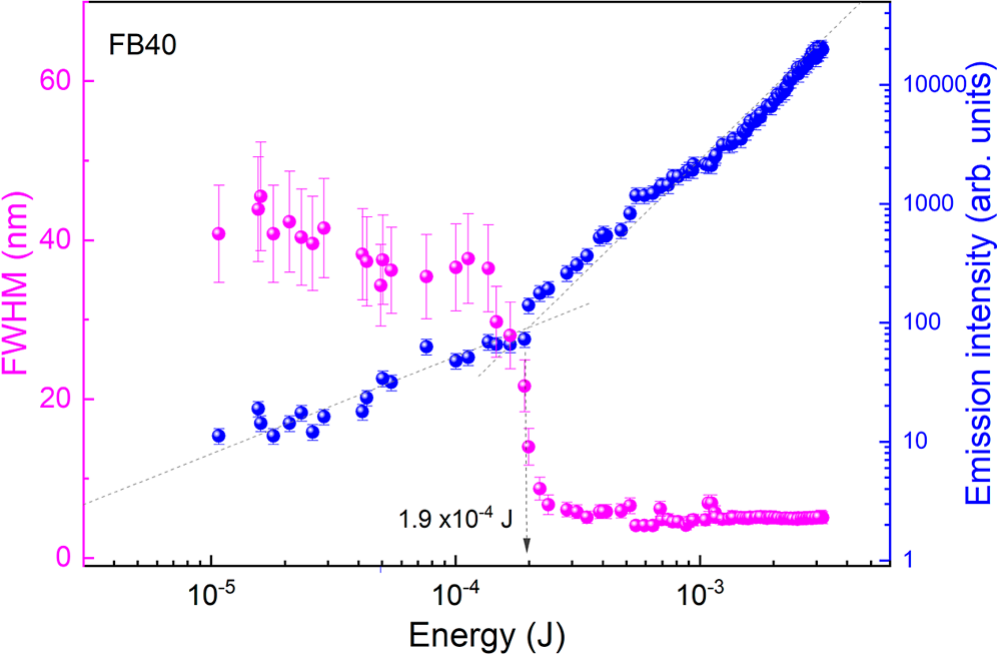
Emission peak as a function of energy pump (blue spheres) and fwhm as a function of energy pump (pink spheres). The dashed lines indicate the laser threshold reached, which is approximately 1.9 × 10^–4^ J for FB40.

Regarding the analysis for the other samples, all six random laser-emitting platforms exhibited a laser energy threshold that varied around 30% and ranged from 1.8 × 10^–4^ J to 2.7 × 10^–4^ J, with fwhm ranging from ∼4 nm to 13 nm (Figure 5). It was observed that both the maximum intensity of random laser emission and the degree of spectral narrowing in the random laser regime were dependent on the quantity of fibers present in the polymeric substrates, which will be discussed later.

**Figure 5:**
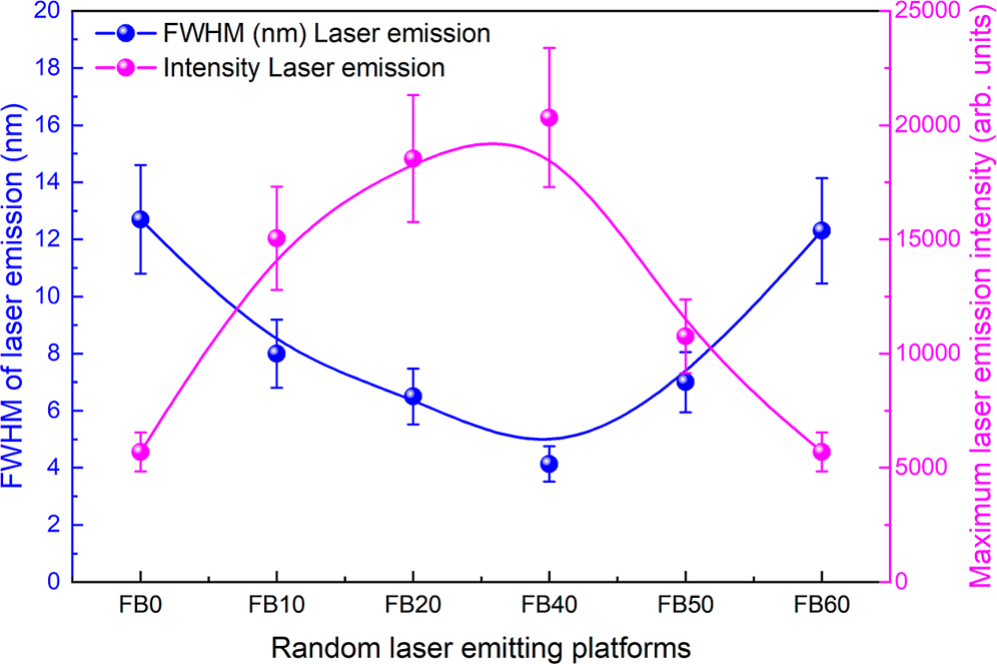
Maximum laser emission intensity (blue spheres) and fwhm (pink spheres) for the distinct samples investigated.

Huang et al.^47^ investigated random lasing action in electrospun nanofibers, confirming a threshold of approximately μJ and an fwhm of 2 nm. Similarly, Oliveira and collaborators^59^ studied random laser emission in dual-sized electrospun fibers, reporting a threshold of 97 μJ with an fwhm around 8 nm. Padiyakkuth^45^ observed a threshold of 175 μJ per pulse and an fwhm of approximately 12 nm for random laser emission in dye-doped electrospun PVDF mats. It is noteworthy that in these studies, the fibers were doped with a dye, whereas in the current work, the fibers were not doped; instead, only the polymeric substrates in which the fibers were embedded were doped. Another interesting result was reported by Romero et al., who studied random lasers using eggshell membranes as the scattering medium.^53^ They observed that although the eggshell exhibited different morphologies and thicknesses between its internal and external layers, the random laser threshold was the same for both the internal and external parts of the eggshell, but the emission intensity was dependent on the external or internal part of the eggshell.

Figure 5 illustrates the behavior of the maximum intensity obtained in the random laser regime and the corresponding fwhm for all of the studied random laser platforms. The result clearly shows that the highest laser emission intensities, reaching ∼20,000 arb. units, were achieved by FB20 and FB40. Interestingly, these two platforms also exhibited the lowest fwhm, around ∼4–7 nm. Additionally, Figure 5 reveals that both FB0 (polymeric substrate without fibers) and FB60 (platform exposed to longer electrospinning time) exhibited similar fwhm and maximum laser intensity (∼13 nm and 15,000 arb. units, respectively).

In the case of FB0, the random laser emission can be attributed to the fact that during photoinitiation, the polymers (SR499 and SR368) can alter their specific size, generating scattering centers. More specifically, because the molecules occupy overlapping spaces, the combined free volumes of the individual molecules exceed the free volume of the resulting polymer. This discrepancy generates internal surface tensions, leading to an uneven surface.^53^ Furthermore, it is reported that the mixture of two polymers (SR368 and SR499) does not result in a miscible solution as the polymers have different viscosity and hardness.^60^ Thus, distinct domains are generated in the polymerized samples. In this way, these distinct domains act as scattering centers for random laser emission.^60^

Figure 5 shows interesting characteristics for the laser emission of platforms prepared with varying electrospinning times. For instance, up to 40 min of electrospinning (FB40), the maximum laser emission intensity increases while the fwhm decreases. However, an opposite behavior occurs for samples FB50 and FB60; that is, as the emission intensity decreases, the fwhm increases. The changes in fwhm and maximum laser emission intensity from 50 min of electrospinning (FB50) onward can be attributed to the quantity of fibers deposited onto the polymer substrate, which act as scattering centers. Up to a specific fiber quantity, corresponding to 40 min of electrospinning, the fiber accumulation leads to a reduction in the fwhm and an increase in maximum laser emission intensity. However, the opposite behavior is observed for times longer than 40 min, where the larger amount of fibers affects the emission intensity, reducing or saturating the maximum laser emission intensity. This can be attributed to the way light propagates depending on the quantity of scattering centers.^11–13,38,61^

Another important factor to consider is the random distribution of fibers onto the polymeric substrates, which can lead to varying emissions. For instance, Sciuti et al.^43^ demonstrated that changing the excitation area influences the random emission. As a result, the intrinsic randomness in fiber deposition, which is typical for electrospinning, can cause differences in random laser emission characteristics such as fwhm, peak emission intensity, and both coherent and incoherent emissions. However, it should be noted that in this study, only incoherent random laser emission was observed, with no coherent emission detected.

Finally, it is important to highlight that, unlike some studies reported in the literature,^1,10,24,26^ in which both the scattering centers and the gain medium are dispersed in an aqueous solution, this work presents a random laser device whose malleability, portability, and flexibility may enhance its potential for future optical and photonic applications. Moreover, it is noteworthy that the fibers were not doped with rhodamine 6G; rather, only the polymeric substrate was, thereby preserving the integrity of the electrospun fibers, which function as effective scattering centers for random lasing.

## 4 Conclusions

This study successfully demonstrated the development of flexible and portable random laser-emitting devices using electrospun fibers integrated into a doped polymeric substrate. By combining rhodamine 6G-doped polymeric substrates with electrospun fibers as scattering centers, it proved to be effective in generating random laser emissions. The resulting platforms exhibited malleability and ease of handling, which are crucial for practical applications. The ability to integrate electrospun fibers onto a flexible polymeric substrate, which is easy to fabricate and manipulate, opens new possibilities for their use in various optical and photonic applications, including in optical sensing and detection tools.

## Supplementary Material



## Notes

Complementary data to the main manuscript, including morphological, structural, and optical characterization of the studied materials; SEM and FEG-SEM images of the polymeric substrate without fibers and at the fiber–substrate interface; FTIR spectra of the neat polymeric substrate (FB0), the composite sample FB60, and neat PAN nanofibers, enabling comparative analysis of their chemical compositions; SEM images of electrospun PAN fibers (FB20) along with the fiber diameter distribution, revealing an average diameter of approximately 800 nm; optical behavior of the samples, including emission spectra under varying pump powers, comparison between spontaneous and stimulated emission regimes, and the evolution of emission peak and fwhm as a function of pump energy, highlighting laser threshold behavior; and experiment aiming to assess the degradation of the samples (PDF)
