# Supplementary material for: Flexible and Portable Random Laser Devices: Integration of Electrospun Fibers and Doped Polymeric Substrates
Source: ACS Omega. 2025 Jul 23;10(30):33288–94. doi: 10.1021/acsomega.5c03313 (PMC12332690; doi:10.1021/acsomega.5c03313)
Supplement: Supplementary file 1 [file ao5c03313_si_001.pdf]

Supporting information

Flexible and Portable Random Laser Devices: Integration of Electrospun Fibers and Doped Polymeric Substrates

Leandro H. Zucolotto Cocca[a,b,c], André L.S. Romero[b], Luiza A. Mercante[d], Kelcilene B. R. Teodoro[c] , Cleber R. Mendonça[b], Leonardo De Boni[b], Daniel S. Correa[c]

[a] Photonics Group, Institute of Physics, Federal University of Goiás, 74690-900, Goiânia, GO, Brazil

[b] Photonics Group, Institute of Physics of São Carlos, University of São Paulo, 13560-970, São Carlos, SP, Brazil

[c] Nanotechnology National Laboratory for Agriculture (LNNA), Embrapa Instrumentação, 13560-970, Sao Carlos, SP, Brazil

[d]Institute of Chemistry, Federal University of Bahia (UFBA) 40170-280, Salvador, BA, Brazil

*Author to whom correspondence should be addressed: leandro.zucolotto@ufg.br

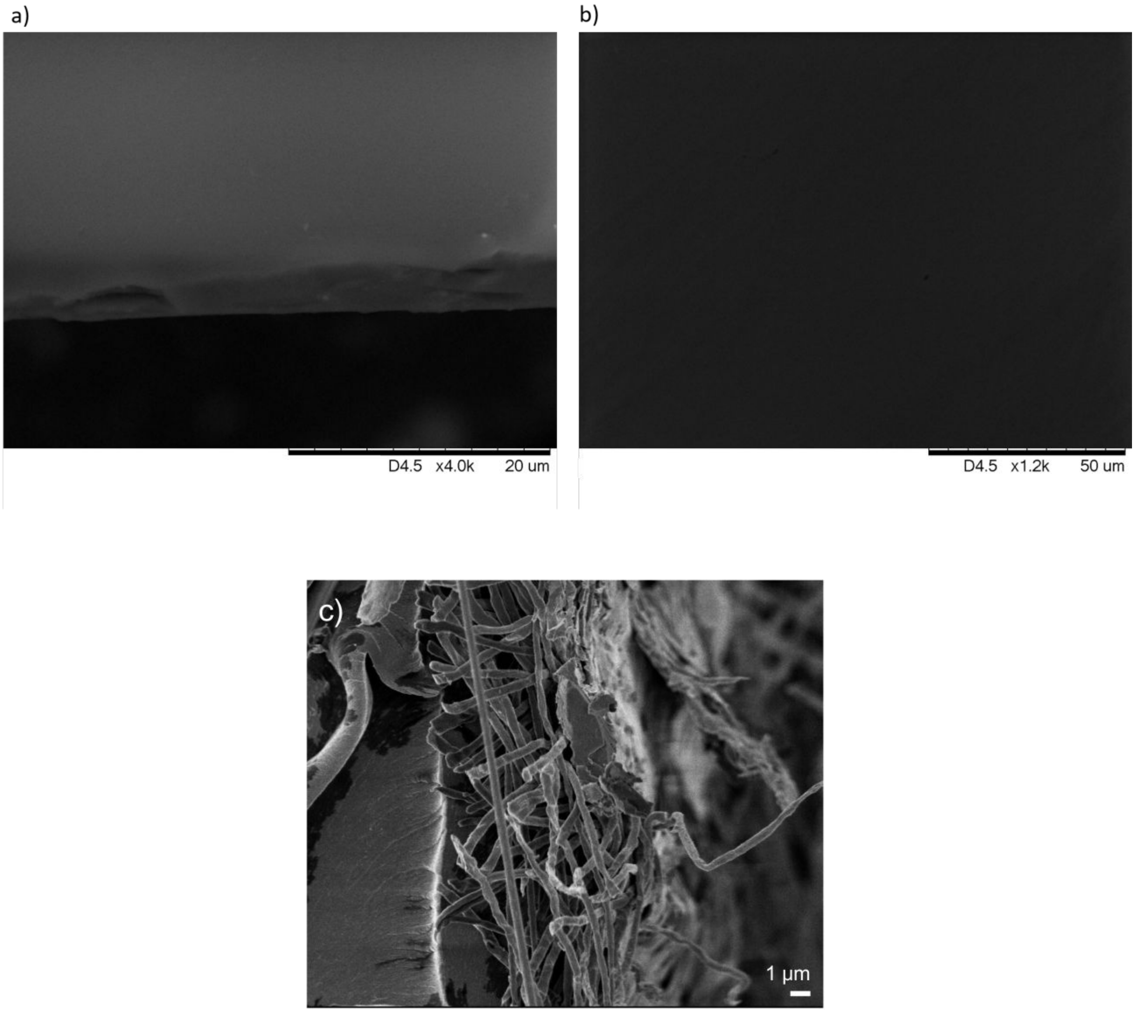

Figure S1: SEM images of the polymeric substrate without fibers. a) Image of the edge of the polymeric substrate. b) Image of the interior of the polymeric substrate without fibers. c) FEG-SEM image of the interface between polymeric substrate and the layer of fibers (sample FB60).

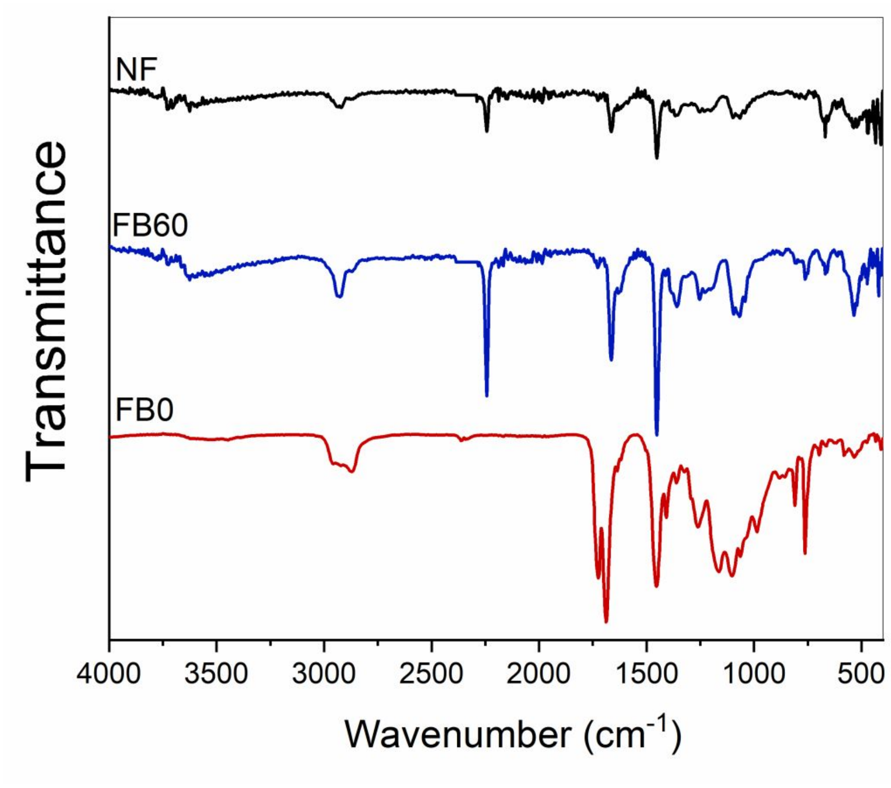

Figure S2 – FTIR spectra recorded to the neat polymeric substrate (FB0), for the sample FB60, and the neat PAN nanofiber.

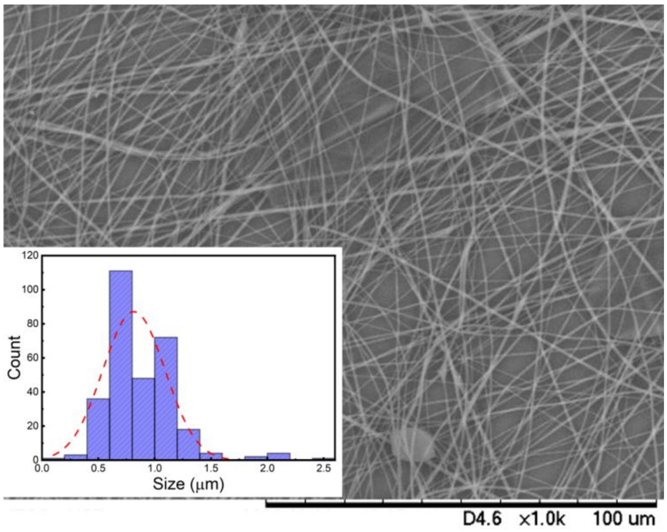

Figure S3: Scanning electron microscope (SEM) images of PAN electrospun fibers (FB20). Inset: distribution of diameters, with average diameter around 800 nm.

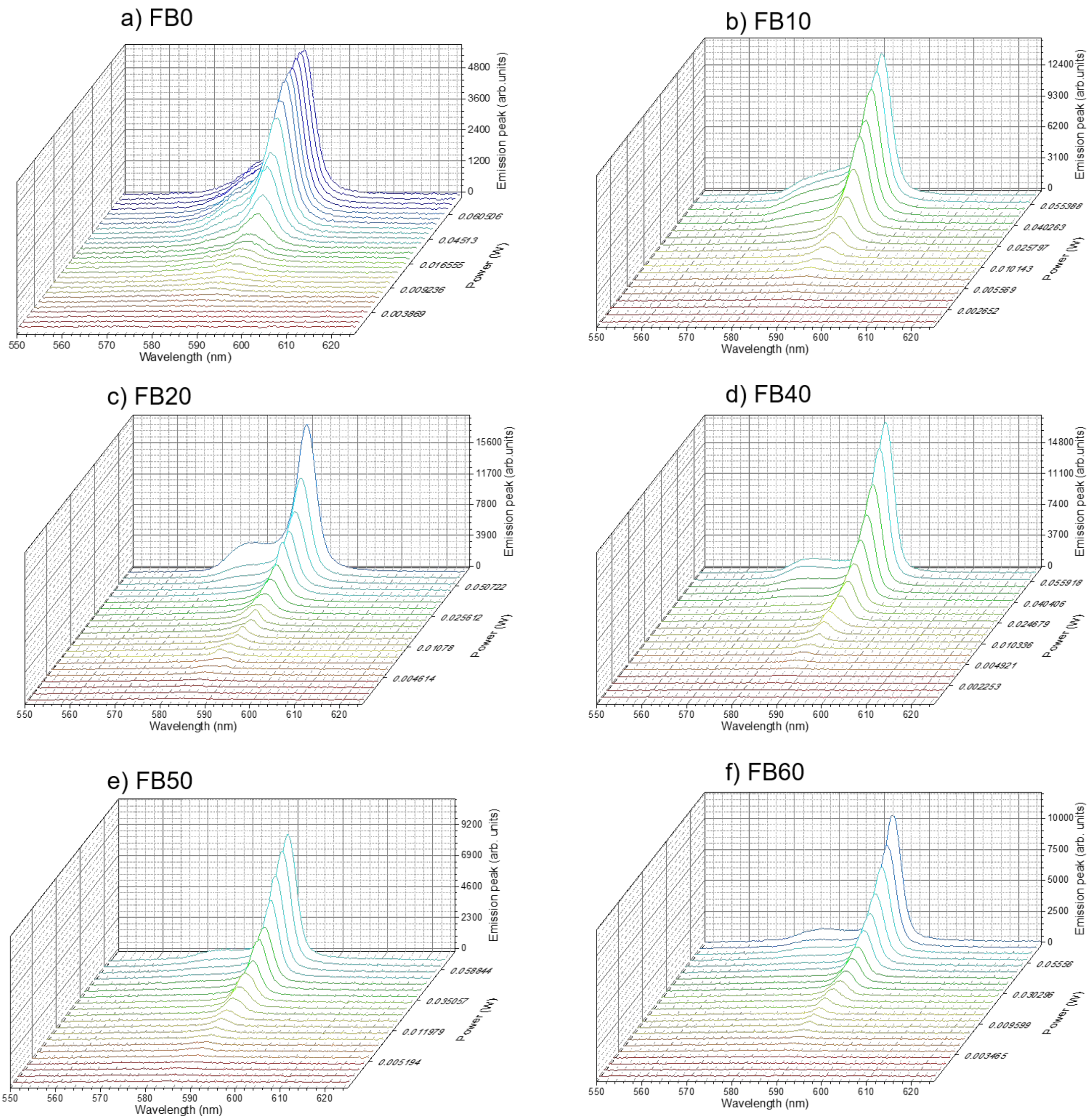

Figure S4. Emission spectra as a function of pump power for samples FB0 (a), FB10 (b), FB20 (c)f FB40 (d), FB50 (e) and FB60 (f).

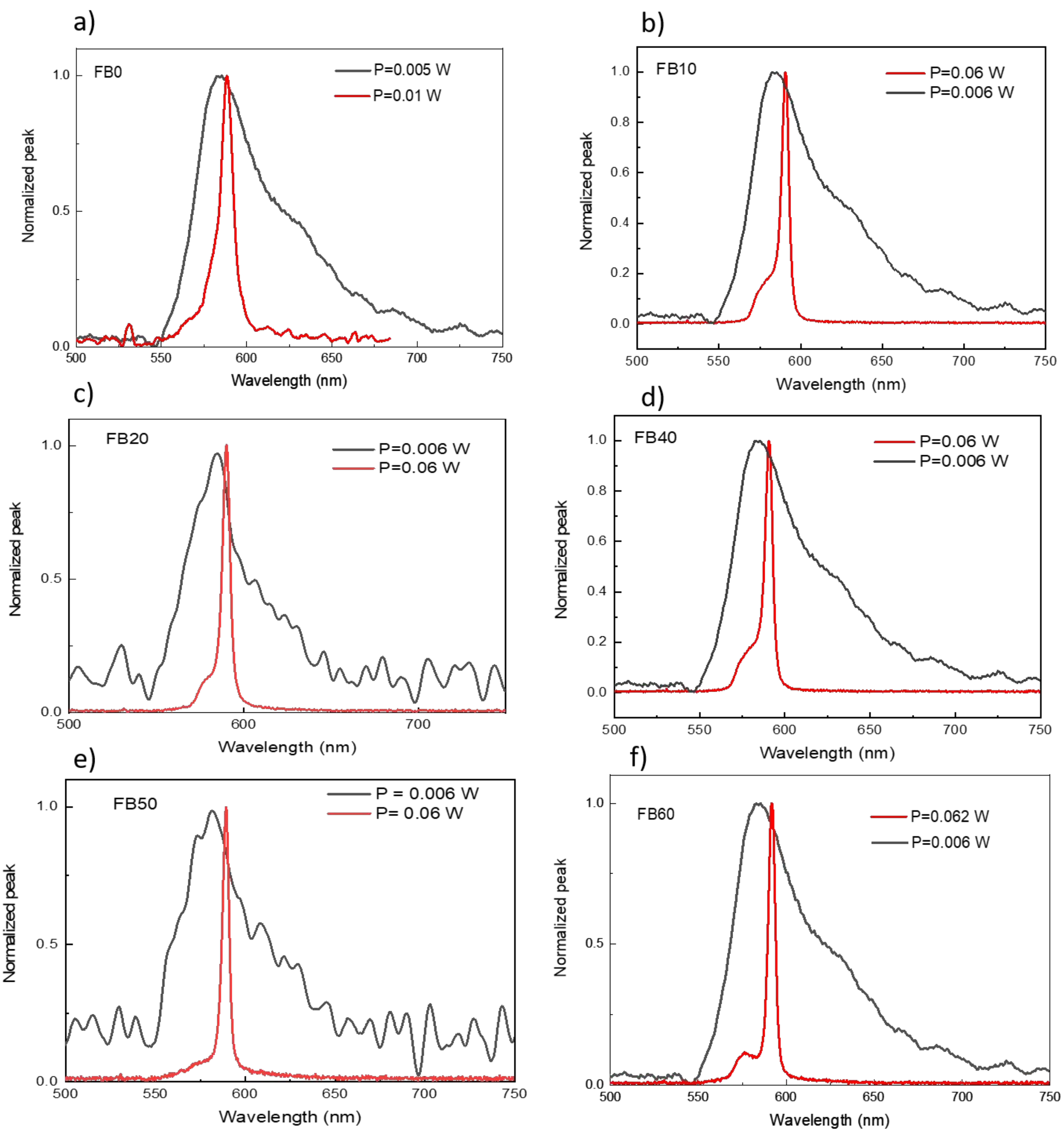

Figure S5) Emission spectrum for FB0 (a), FB10 (b), FB20 (c), FB40 (d), FB50 (e) and FB60 (f). The black line represents the emission spectrum under low excitation power (fluorescence/spontaneous emission regime) and the red line corresponds to the emission spectrum when the platform is excited with laser powers exceeding the laser threshold (laser regime).

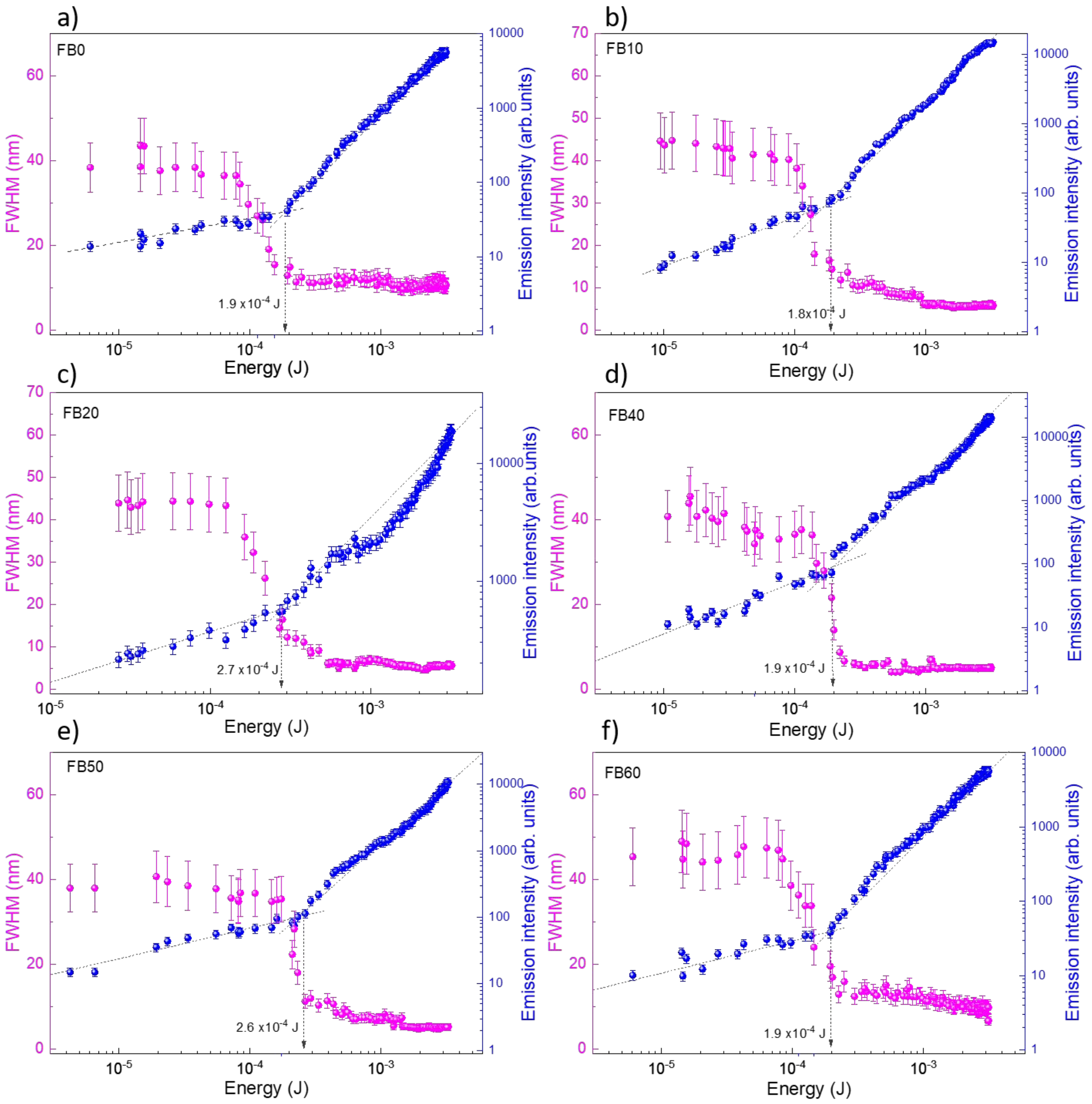

Figure S6) Emission peak as a function of energy pump (blue spheres) and FWHM as a function of energy pump (pink spheres) for FB0 (a), FB10 (b), FB20 (c), FB40 (d), FB50 (e) and FB60 (f). The dashed lines indicate the laser threshold reached.

Degradation tests

The samples were subjected to degradation tests caused by the excitation (pump) laser. These can further influence the performance and stability of random laser emission in the long term[1]. Cumulative damage results because of repeated interaction

between the sample and laser pulses, directly affecting such emission characteristics as the peak value and the full width at half maximum (FWHM) of the emission spectrum[1].

To determine the impact of material degradation due to exposure to the laser, a controlled experiment was carried out with the application of flexible polymeric substrates exposed to differing counts of pump laser pulses. The same experiment setup outlined earlier was utilized with the laser pump power held constant at 60 mW, which is the maximum energy power utilized in the random laser experiments. Peak intensity and FWHM spectral broadening were tracked as a function of the number of pump pulses with a spectrometer operated on 1-second integration time. All emission spectra were taken throughout a total measurement time of 250 seconds, allowing dynamic measures of the emission profile with respect to time.

Figure S7 shows the tests of degradation analysis in terms of the percent change in peak intensity of the random laser emission and spectral FWHM as a function of pump pulse number. The purpose of this test was to assess the degradation of the sample; therefore, the irradiation was performed using the highest power level employed in the experiments (60mW). As expected, prolonged exposure to a large number of pulses leads to degradation of the polymer substrate, as evidenced by variations in both the FWHM and peak intensity of the emission. However, for up to 500–1000 pulses, the degradation remains below 15 – 20%, based on these same metrics. It is also important to highlight that these tests were carried out using the maximum power applied in the experiments, although in the actual measurements only a single acquisition is performed.

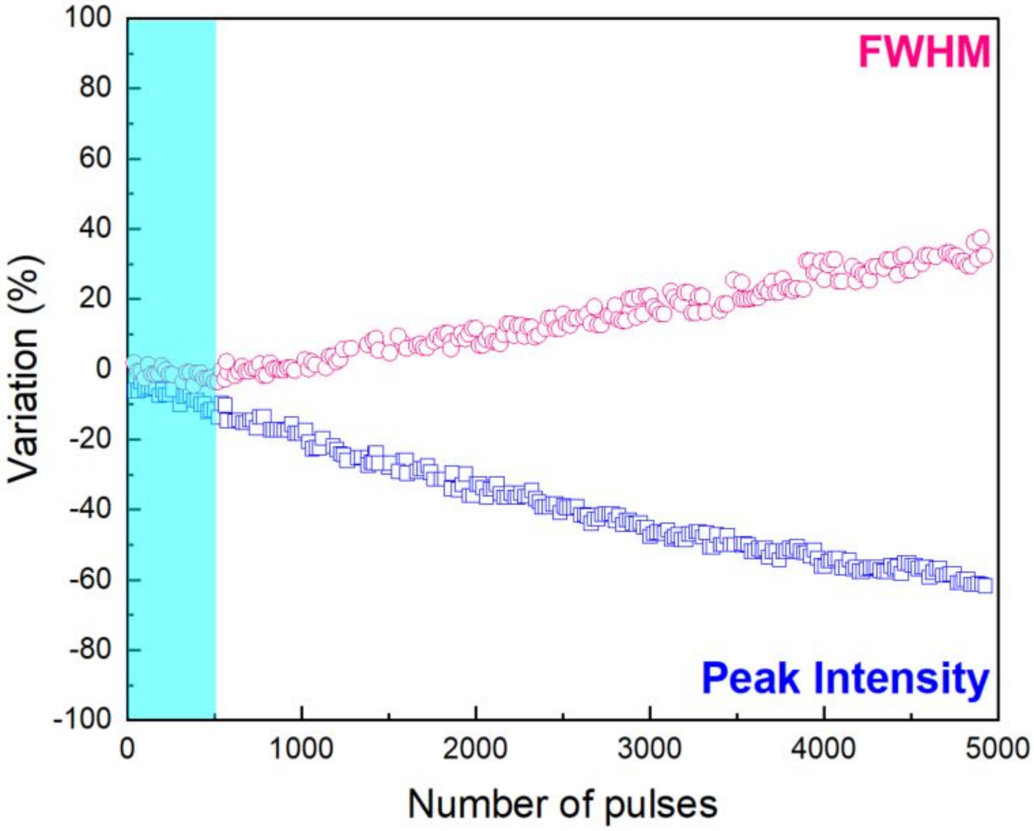

Figure S7: Degradation effects measured by changes (%) in the values of peak intensity (blue square) and FWHM (pink circles) of the random laser as a function of the number of pump laser pulses.

References

[1] Romero, A. L. S., Gonçalves, T. S. & De Boni, L. Combining eggshell membrane biomaterial and polymeric film as a platform for random laser applications. *J Lumin* **252**, 119369 (2022).
